# *Meloidogyne paramali* n. sp. (Nematoda: Meloidogyninae) and First Report of *M. marylandi* in maple and yacca tree from Japan

**DOI:** 10.2478/jofnem-2022-0036

**Published:** 2023-04-19

**Authors:** Jianfeng Gu, Yiwu Fang, Xinxin Ma, Baolin Shao, Kan Zhuo

**Affiliations:** Ningbo Key Laboratory of Port Biological and Food Safety Testing, Ningbo Customs Technology Center (Ningbo Inspection and Quarantine Science Technology Academy), Ningbo, Zhejiang 315100, P.R. China; Ningbo Zhongsheng Product Testing Co., Ltd, Ningbo, Zhejiang 315100, P.R. China; Technical Center of Chengdu Customs, Chengdu, Sichuan 610041, P.R. China; Laboratory of Plant Nematology, South China Agricultural University, Guangzhou, Guangdong 510642, P.R. China

**Keywords:** molecular, new species, phylogeny, rDNA, taxonomy, root-knot nematode

## Abstract

*Meloidogyne paramali* n. sp. was detected from Japanese maple trees (*Acer palmatum*) from Chiba, Japan during quarantine inspections in China. This species is characterized by second-stage juveniles (J2) with short tail length 32.2 (24–36.8) μm, finely rounded to broadly pointed tail terminus with extremely short hyaline tail terminus 4.3 (3.0–4.9) μm; perineal patterns of females characterized by an oval or irregular appearance, with round and low dorsal arch, and fine and smooth striae. *M. paramali* n. sp. is very similar to *M. mali* in that the perineal pattern has fine, smooth striae and both J2 have a short tail, but it can be distinguished from the latter by perineal pattern of the female (lateral field distinct *vs*. indistinct), shorter J2 hyaline tail terminus (4.3 [3.0–4.9] μm *vs*. 8.2 [4.8–12.7] μm, and by J2 tail with finely rounded to broadly pointed tail terminus, never sharply pointed *vs*. finely rounded and almost pointed. The polytomous key codes of the new species are as follows: *Female*: A21, B2, C32, D4; *Male*: A21, B3, C2, D1, E2, F2; *J2*: A2, B23, C43, D34, E12, F34. Detailed phylogenetic analysis based on partial 18S, ITS, D2-D3 28S, and partial mtCOI sequences also confirmed it as a new species, which is very close to *M. mali* and *M. vitis* and forms molecular group VIII. *M. marylandi* and other *Meloidogyne* species detected from plants from Japan in China are also discussed.

Root-knot nematode (*Meloidogyne* spp.) is one of the most important polyphagous pests in agriculture and the most devastating pathogens of crops (Perry et al., 2009). Infestation crops greatly impact plant health, yield, and quality. They have been adapted to parasitize on a large number of plants and over 3,000 wild and cultivated plant species are reported to be affected (Hussey and Janssen, 2002). They are distributed worldwide over a wide range of geographical conditions of tropical, subtropical, and temperate regions.

Perry et al. (2009) listed 97 nominal species. Ahmed et al. (2013) synonymized *Meloidogyne ulmi* Palmisano and Ambrogioni, 2000 to *M. mali* Itoh et al., 1969, and *M. dimocarpus* Liu and Zhang, 2001 was missing in this book. Subsequently, *M. lopezi* Humphreys-Pereira, Flores-Chaves, Gómez, Salazar, Gómez-Alpízar & Elling, 2014, *M. pakistanica* Shahina, Nasira, Salma, Mehreen & Bhatti, 2015, *M. spartelensis* Ali, Tavoillot, Mateille, Chapuis, Besnard, Bakkali, Cantalapiedra-Navarrete, Liébanas, Castillo & Palomares-Rius, 2015, *M. aberrans* Tao, Xu, Yuan, Wang, Lin, Zhuo & Liao, 2017, *M. daklakensis* Trinh, Le, Nguyen, Nguyen, Liebanas & Nguyen, 2018, *M. moensi* Le, Nguyen, Nguyen, Liebanas, Nguyen & Trinh, 2019, *M. aegracyperi* Eisenback, Holland, Schroeder, Thomas, Beacham, Hanson, Paes-Takahashi & Vieira, 2019, and *M. vitis* Yang, Hu, Liu, Chen, Peng, Wang & Zhang, 2021, have been reported. Ghaderi and Karssen (2020) listed 104 valid species. More recently, Subbotin et al. (2021) listed only 98 nominal species; *M. actinidiae* Li and Yu, 1991, *M. dimocarpus*, *M. hainanensis* Liao and Feng, 1995, *M. lini* Yang, Hu & Xu, 1988, *M. decalineata* Whitehead, 1968, *M. oteifae* Elmiligy, 1968, and *M. pakistanica* are no longer considered as valid species.

In May 2018, during quarantine inspections of imported plants in Ningbo Port, China, around 100 second-stage juveniles (J2) of root-knot nematodes were extracted from one of five Japanese maple trees (*Acer palmatum* Thunb f.), with a little soil and medium surrounding the roots, using a modified Baermann funnel. Females and males were found later. The specimens appeared very similar to *M. mali*, but some differences were noted. Further detailed morphological and molecular characterization confirmed the status of this population as a new species and it is described and illustrated in the present study as *M. paramali* n. sp.

Later, in July 2020, *M. marylandi* Jepson and Golden, 1987 was detected in the soil and medium soil surrounding the roots of *Podocarpus*, although, since only tens of juveniles were detected, it remains to be determined whether *Podocarpus* is the host plant.

In both cases, no symptoms of infection on trees were detected. The research reported here provides a detailed account of morphological and molecular study of a new *Meloidogyne* species, which is proposed in the present study as *M. paramali* n. sp. from Japanese maple and *M. marylandi* from the rhizospere of *Podocarpus* from Japan.

## Materials and methods

### Isolation and morphological observation of nematodes

Rhizospheric soil and medium soil samples of maple and *Podocarpus* trees from Japan were obtained by making two to three holes, each having a depth of about 20 cm to 30 cm, and about 1 kg of soil mixed with medium soil and some roots were collected. Nematodes were extracted with a Baermann funnel for 24 hr to 48 hr. After extraction of J2, the roots were washed and observed with a dissecting microscope, and females and males were recovered. For preparation of light microscopy (LM), males and J2s were relaxed with gentle heat, fixed in a solution of 4% formaldehyde, and processed using the glycerin–ethanol method. Perineal patterns of mature females were prepared as described previously (Hartman et al., 1985). The perineal pattern was trimmed and transferred to a drop of glycerin for observation. Nematodes were measured and photographed with a Zeiss Imager Z1 microscope equipped with a Zeiss AxioCam MRm CCD camera (Oberkochen, Germany).

### Molecular analyses

DNA samples were obtained from freshly extracted nematodes. Briefly, two repeats of each single J2 individual, one male and one female, were transferred separately to a PCR tube containing 8 μL ddH_2_O and 1 μL 10× PCR buffer. The tubes were stored at −70°C for at least 2 hr, and then pipetted 1 μL proteinase K (100 μg/mL) was introduced into the solution containing the melted remains of the nematodes. Subsequently, the tubes were incubated at 65°C for 1 hr to 2 hr and the proteinase K was denatured at 95°C for 10 min. Four sets of primers (synthesized by Invitrogen [Shanghai, China]) were used in the PCR analyses to amplify partial 18S, full length ITS, and partial 28S D2-D3 regions of rDNA. The 18S region was amplified with one pair of primers (forward primer 988F, reverse primer 1912R) and then another pair (1813F and 2646R) (Holterman et al., 2006), the ITS region was amplified with the forward primer TW81 (Ferris et al., 1993) and the reverse primer AB28 (Vrain, 1993), and the 28S D2-D3 region was amplified with the forward primer D2A and the reverse primer D3B (De Ley et al., 1999). The intergenic region between the cytochrome oxidase subunit II (CO II) and 16S rRNA (lrRNA) genes was amplified with forward primer C2F3 and reverse primer MRH106 (Stanton et al., 1997). For *M. marylandi*, only full length ITS and partial 28S D2–D3 regions of rDNA were amplified. PCR products were separated on 1% agarose gels and visualized by staining with ethidium bromide. Positive PCR products were cloned with the pMD 18-T Vector Cloning Kit (Promega, Beijing, China) using TOP10 competent cells following the manufacturer’s instructions before sequencing. PCR products were sequenced in both directions with PCR primers in Invitrogen.

The sequences of partial 18S, full length ITS, 28S D2-D3 region, and intergenic region between CO II and 16S rDNA of *M. paramali* n. sp. were compared with other relevant *Meloidogyne* sequences available with GenBank. The selected sequences were aligned by MAFFT v.7.205 (Katoh and Standley 2013) with default parameters. The alignments of sequences were manually edited and assembled in one dataset by using AliView (Larsson, 2014). The best-fitted model of DNA evolution was obtained using jModelTest2 (Darriba et al., 2012) with the Akaike information criterion (AIC). The SYM + I + G model was selected for 18S region partition, the TVM + I + G model was selected for 28S D2–D3 region partitions, and the GTR + G model was selected for full length ITS and intergenic region between CO II and 16S rDNA.

The Bayesian concatenated tree was inferred using MrBayes 3.2.3 (Ronquist and Huelsenbeck, 2003) with four chains (three heated and one cold). Model parameters were unlinked and the overall rate was allowed to vary across partitions. The number of generations for the total analysis was set to 3 × 107, with the chains sampled every 1,000 generations and the burn-in value set to 25%. The Markov chain Monte Carlo (MCMC) method within a Bayesian framework was used to estimate the posterior probabilities of the phylogenetic trees using the 50% majority rule (Larget and Simon, 1999). The consensus tree was selected to represent the phylogenetic relationships with branch length and support level and visualized using TreeGraph 2 (Stöver and Müller, 2010).

## Nomenclatural acts

The electronic edition of this article conformed to the requirements of the amended International Code of Zoological Nomenclature, and hence the new names used in this text are available under that Code from the electronic edition of this article. This published work and the nomenclatural acts it contains have been registered in ZooBank, the online registration system for the ICZN. The ZooBank Life Science Identifiers (LSIDs) can be resolved and the associated information viewed through any standard web browser by appending the LSID to the prefix “http://zoobank.org/”. The LSID for this publication is: urn:lsid:zoobank.org:pub:83E862FE-A541-48D4-A605-6D6CBCDA4531. The electronic edition of this work was published in a journal with an ISSN, and has been archived and is available from the following digital repositories: PubMed Central, LOCKSS.

## Results

### Meloidogyne paramali^*^ n. sp.

(Figs. 1–4)

**Figure 1:**
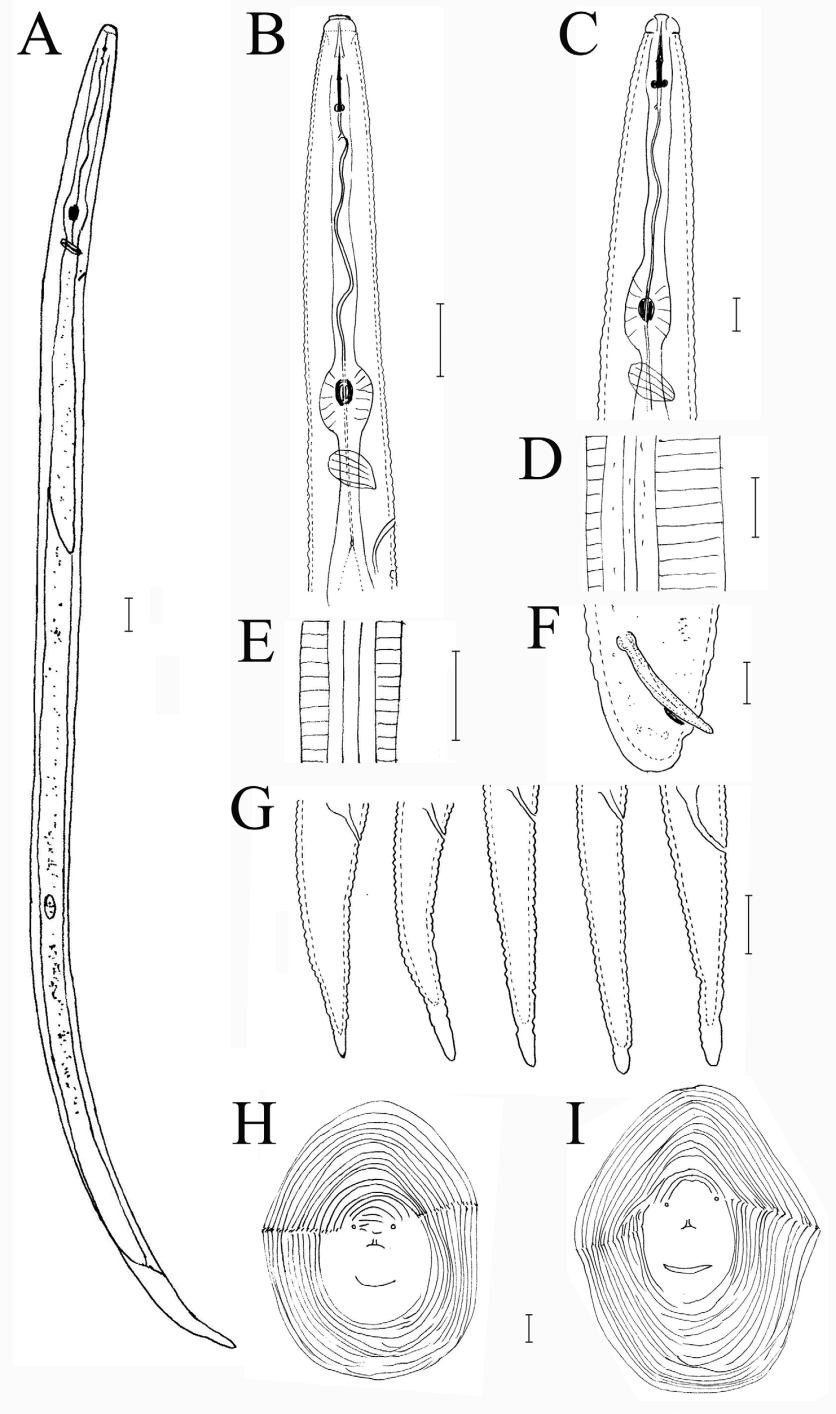
*Meloidogyne paramali* n. sp. A: J2; B: Anterior region of J2; C: Anterior region of male; D: Lateral region of male; E: Lateral region of J2; F: Male tail region; G: Variations of J2 tail; H, I: Female perineal patterns. (Scale bars = 10 μm).

### Measurements

Measurements of J2 are available in Table 1.

**Table 1:** Morphometrics of J2 of Meloidogyne paramali n. sp and M. mali. All measurements are in micrometer and in the form: mean ± s.d. (range).

		Meloidogyne paramali n. sp	M. mali detected from Japanese maple tree^a^
	Holotype J2	Paratype J2s	-
*n*		20	20
Body length	441	433 ± 13.1 (402–455)	425 ± 30.1 (362–466)
Body width	14.2	13.7 ± 0.8 (12.5–15.3)	14.0 ± 1.1 (13.1–18.1)
Head end to metacorpus valve	60.2	58.6 ± 2.2 (54–64)	53.5 ± 2.1 (49.5–56.8)
Head end to excretory pore	80.6	76.4 ± 3.6 (64–80.8)	74.1 ± 4.2 (68.8–82.3)
Head width	5.7	5.6 ± 0.2 (5.0–5.9)	5.1 ± 0.41 (4.1–5.7)
Head height	3.2	3.0 ± 0.3 (2.2–3.5)	2.6 ± 0.32 (2.1–3.2)
Stylet length	11.8	11.7 ± 0.5 (10.8–12.5)	10.5 ± 0.5 (9.5–11.6)
Stylet conus	6.5	6.4 ± 0.3 (6.0–7.0)	5.7 ± 0.5 (4.7–6.7)
Knobs height	1.1	1.1 ± 0.1 (0.9–1.2)	1.2 ± 0.2 (0.8–1.6)
Knobs width	2.0	1.8 ± 0.3 (1.5–2.4)	2.1 ± 0.2 (1.8–2.7)
DGO	3.6	3.3 ± 0.3 (2.4–3.9)	4.4 ± 0.57 (3.5–5.5)
Tail length	33.6	32.2 ± 3.5 (24–36.8)	32.7 ± 3.0 (29.2–39.3)
Anal body diam.	8.2	8.0 ± 0.6 (6.9–8.7)	7.9 ± 0.9 (5.9–9.6)
Tail hyaline portion	4.3	4.3 ± 0.6 (3.0–4.9)	7.2 ± 2.3 (3.9–9.3)
a	31.0	31.7 ± 1.6 (28.2–34.8)	30.4 ± 2.6 (25.2–34.5)
b(body length/Head end to metacorpus valve)	7.3	7.4 ± 0.2 (7.0–7.7)	8.0 ± 0.6 (6.8–9.2)
c	13.1	13.6 ± 1.9 (11.7–18.9)	13.2 ± 1.1 (11.6–15.3)
c’	4.1	4.1 ± 0.5 (2.8–4.8)	4.2 ± 0.7 (3.1–5.6)
m	55.0	54.1 ± 2.9 (50.5–58.9)	54.5 ± 3.8 (47–60.3)
O	30.5	28.1 ± 3.7 (21.4–34.5)	41.9 ± 5.8 (31.6–53.9)
Head width/head height	1.8	1.87 ± 0.2 (1.7–2.5)	2.0 ± 0.2 (1.6–2.3)
h% (Tail hyaline portion/tail length×100)	12.8	13.7 ± 1.8 (9.8–17.1)	21.7 ± 5.9 (12.7–27.8)

aGu et al., 2013.

### Description

*Female (n = 5)*

Females were rare, and only 5 could be measured. The measured parameters are the following – length (including neck): 820.6 ± 82.0 (710–962) μm; length of neck: 166.0 ± 19.4 (135–190) μm; body width: 737.2 ± 72.8 (630–849) μm; DGO from stylet base: 5.6 ± 0.8 (4.7–6.5) μm; and stylet length: 14.5 ± 1.5 (12.5–16.9) μm.

Females were completely enclosed by gall tissue. Their bodies were translucent-white, variable in size, and ovoid to pear-shaped. The neck was sometimes prominent. The body cuticle was distinctly annulated, and annuli were smaller in the anterior neck region. The head region was set off from body and the cephalic framework was weakly sclerotized, with distinct head caps and indistinct annulations. The stylet cone was slender and slightly curved; the shaft was observed to have a gradually wider shape posteriorly near the junction, with knobs. The knobs were well developed, round to transversely ovoid, and slightly concave anteriorly. The excretory pore was located posterior to stylet base. The distance of dorsal pharyngeal gland orifice to stylet base was approximately 5 μm to 7 μm. Perineal patterns were oval or irregular, with distinct lateral lines; dorsal arches were often round, and dorsal and ventral striae smooth. A dorsal arch with shoulders was sometimes present, formed by a slight indentation of the dorsal striae near the lateral lines. The anus was within a cuticlar fold, and the phasmids were conspicuous. Inter-phasmidal distance (22.2 [18.9–28.7] μm) was wider than the length of vulval slit (17.3 [16.1– 18.3] μm), the distance of the vulva from an imaginary line joining the phasmids was 25.3 (24.0–26.9) μm, and the distance between the vulva and anus was 16.3 (12.4–19.1) μm.

### Male (n = 2)

Males are rare, and only two were recovered. Their characteristics are the following – body length: 1,545 μm and 1,819 μm; body width: 32.1 μm and

* Specific epithet is derived from its similarity to M. mali. 47.0 μm; a: 48.1 and 38.7; stylet length: 18.9 μm and 20.1 μm; DGO: 6.5 μm and 7.0 μm; spicules’ length: 31 μm and 33 μm; head to the middle of valve plate: 93 μm and 99 μm; and tail length: 9.8 μm and 11.4 μm.

**Figure 2:**
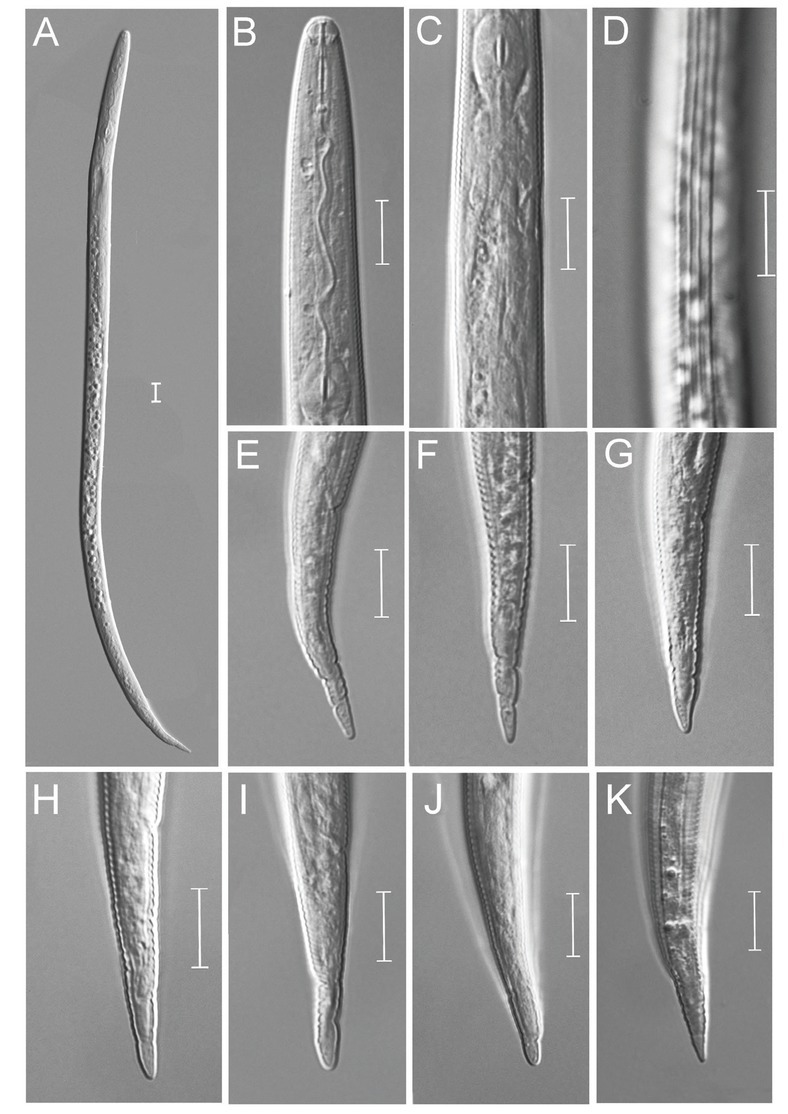
Light photomicrographs of *Meloidogyne paramali* n. sp. J2. A: Entire body; B: Anterior region; C: Post median bulb region; D: Lateral region; E–K: Tail region. (Scale bars = 10 μm).

The bodies were vermiform, tapering anteriorly, bluntly rounded posteriorly twisting through 90°, and the tails were bluntly rounded. Body annuli were large, distinct, and 2.4 μm to 2.9 μm in width. The lateral field was with four lines in the mid body region, beginning as two lines at about the stylet base and ending as two or three lines at the tail tip. The cephalic framework was moderately developed, and vestibule extensions were distinct. The head was set off from the body, without labial annulus. The stylet was robust, with rounded basal knobs. The cone was slightly longer than the shaft. The shaft was cylindrical, widening slightly near the junction, with knobs. The dorsal pharyngeal gland orifice branched into three channels, with distinct ampulla. The procorpus was distinct, while the median bulb was ovoid. Pharyngo-intestinal junction occurred at the level of nerve ring, and was indistinct. Three nuclei were present in gland lobe. The testis was outstretched or anteriorly reflexed. Spicules were arcuate, and gubernaculum distinct. The tail was short with broadly rounded terminus, with indistinct phasmids.

**Figure 3:**
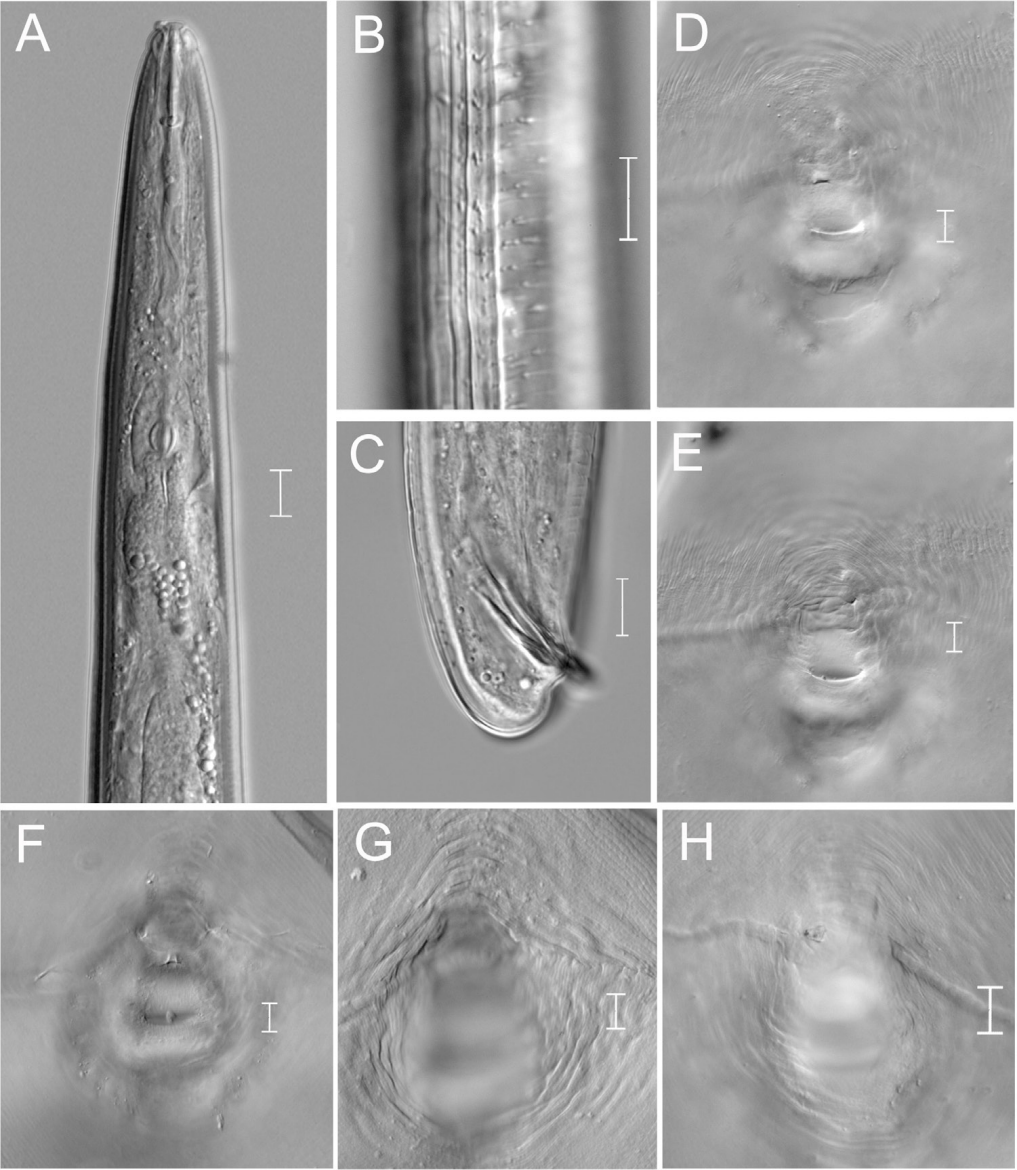
Light photomicrographs of *Meloidogyne paramali* n. sp. male and female. A: Male head region; B: Male lateral region; C: Male tail region; D–H: Female perineal patterns. (Scale bars = 10 μm).

**Figure 4:**
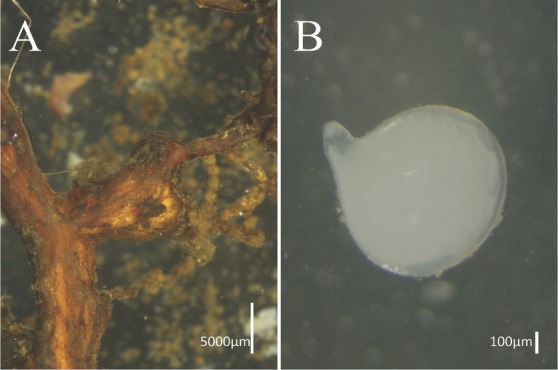
Light photomicrographs of host plant roots infected by *Meloidogyne paramali* n. sp. and female. A: Root-knot; B: Female.

### Second-stage juvenile (J2)

About 50 J2s were observed, and 20 of them were measured. The bodies were observed to be vermiform, clearly annulated, and straight or slightly ventrally bent near the tail region. The anterior ends were truncated, with the head region only slightly set off from the body. The lateral region was slightly larger than one-third of the body width, with four lines. The stylet was delicate, and the cone was straight, narrow, and sharply pointed; the shaft was observed to become slightly wider posteriorly, while the knobs were small and rounded. The procorpus was faint, while the metacorpus was oval-shaped with an enlarged lumen lining. The isthmus was clearly defined, while the pharyngo-intestinal junction was difficult to observe. The gland lobe variable in length, and it ventrally overlapped the intestine. The excretory pore was distinct, and was located 64 μm to 81 μm from the head, while the hemizonid was three to five annuli posterior to the excretory pore. The tail was conoid, with finely rounded to broad pointed terminus, but never sharply pointed. The hyaline tail terminus was very short (4.3 [3.0–4.9] μm), and indistinct. The phasmids were small, difficult to observe, and located posterior to the anus.

### Eggs

The eggs had a morphology that is typical for this genus. The egg masses were usually protruding from root tissues on the roots.

### Type habitat and locality

The nematodes in question were isolated from Japanese maple (*Acer palmatum* Thunb.) imported from Chiba, Japan and inspected in Ningbo Port, P.R. China during May, 2018.

### Type material

To earmark the necessary holotype female, five females, two males, and 26 second-stage juvenile paratypes (slide number 5445-1~5445-10) have been deposited at the nematode collection of Ningbo Customs Technology Center, China. Additionally, five paratype J2s (slide numbers T620) are deposited in the Canadian National Collection of Nematodes, Ottawa, Canada.

### Diagnosis and relationships

*Meloidogyne paramali* n. sp. is characterized by J2 with a short tail length of 32.2 (24–36.8) μm, finely rounded to broad pointed tail terminus, and an extremely short hyaline tail terminus of 4.3 (3.0–4.9) μm. The perineal pattern of females was oval or irregular, with round dorsal arch, distinct lateral lines, and fine and smooth striae. The polytomous key codes of the new species according to Subbotin et al. (2021) are: *Female*: A21, B2, C32, D4; *Male*: A21, B3, C2, D1, E2, F2; *J2*: A2, B23, C43, D34, E12, F34.

Based on the perineal pattern of females (A4) and the similar J2 tail length (C43), we may infer that *Meloidogyne paramali* n. sp. is very similar to *M. mali* and *M. nataliei* Golden et al., 1981. The new species can be distinguished from *M. mali* by the perineal pattern, which has a distinct lateral field; additionally, the hyaline terminus of the J2 tail is shorter (4.3 [3.0– 4.9] μm *vs*. 8.2 [4.8–12.7] μm), and the tail is finely rounded to broadly pointed in *M. paramali* n. sp., but it is never sharply pointed as it is in *M. mali*.

It differs from *M. nataliei* by shorter J2 body length (433 [402–455] μm *vs*. 599 [539–641] μm), shorter J2 stylet length (11.7 [10.8–12.5] μm *vs*. 22.4 [21.9–22.8] μm), shorter tail hyaline portion (4.3 [3.0–4.9] μm *vs*. 8.4 [7.0–11.0] μm), and much smaller c value (13.6 [11.7–18.9] *vs*. 22 [19–26]).

*M. caraganae* Shagalina et al., 1985, *M. coffeicola* Lordello and Zamith, 1960, *M. kikuyensis* De Grisse, 1961, *M. nataliei*, *M. suginamiensis* Toida and Yaegashi, 1984, *M. turkestanica* Shagalina et al., 1985, and *M. vandervegtei* Kleynhans, 1988 have similar J2 tail length (C4 or 43), but the perineal patterns are different: D3 for *M. caraganae*, *M. suginamiensis*, *M. turkestanica* and *M. vandervegtei*, D2 for *M. coffeicola*, and D5 for *M. kikuyensis*.

In the phylogenetic trees (Figs. 6–9), *M. vitis*, *M. paramali* n. sp., and *M. mali* formed a well-separated clade, which is named molecular group VIII (Subbotin et al., 2021). *Meloidogyne paramali* n. sp. differs from *M. vitis* in that the J2 tail is shorter (32.2 [24–36.8] μm *vs*. 57.43 [47.01–63.77] μm), and the J2 c value is bigger (13.6 [11.7–18.9] *vs*. 6.95 [6.15–8.77]).

**Figure 5:**
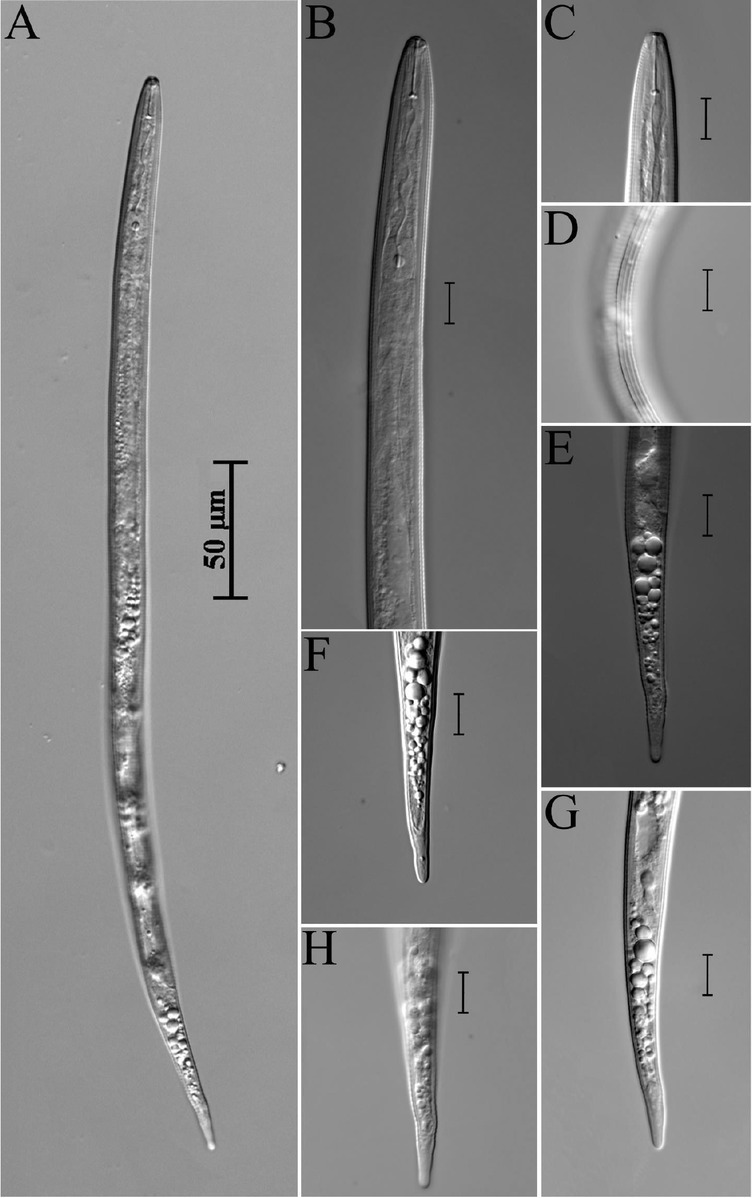
Light photomicrographs of *Meloidogyne marylandi* J2. A: Entire body; B, C: Anterior region; D: Lateral region; E–H: Tail region. (Scale bars = 10 μm).

**Figure 6:**
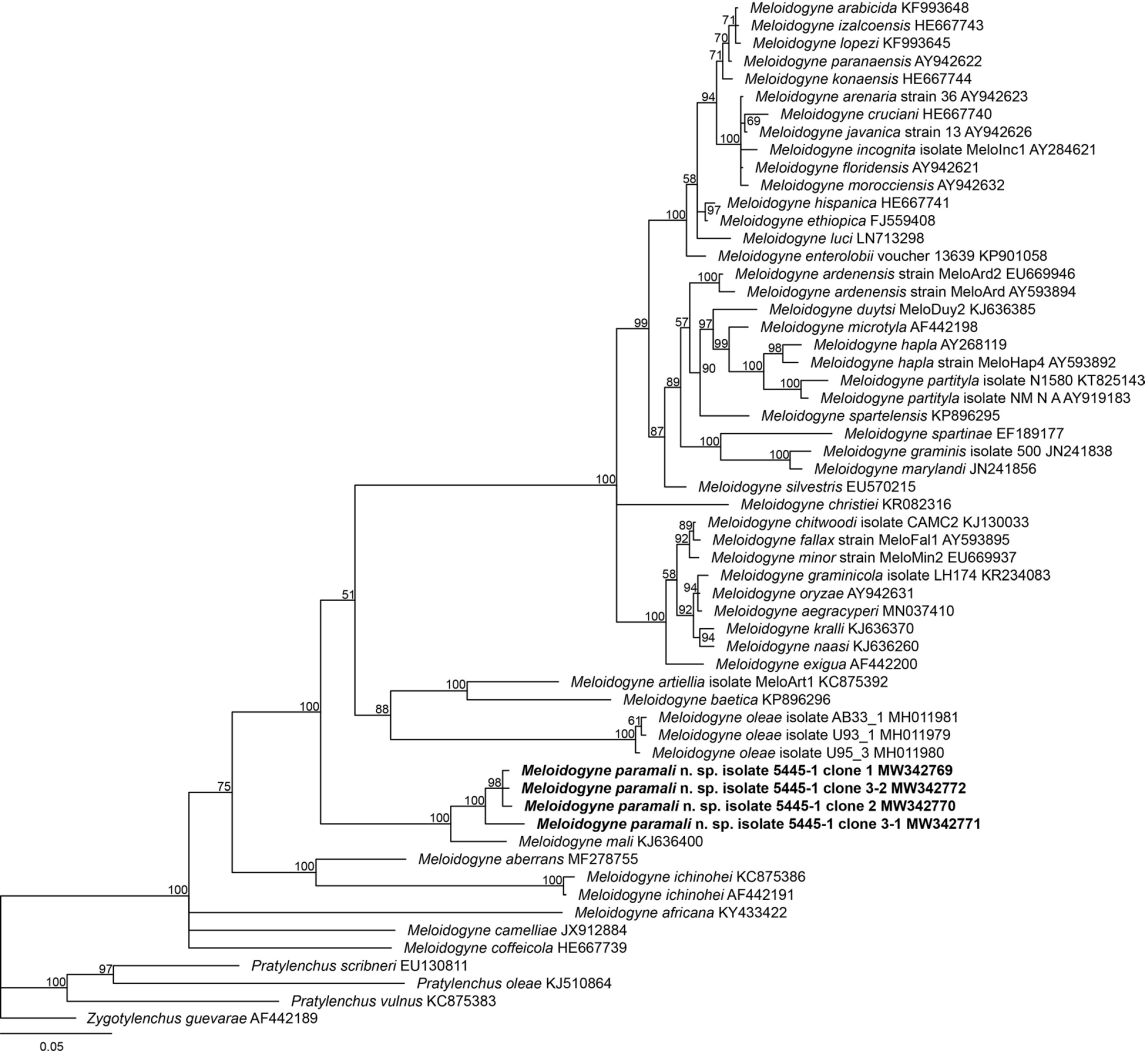
Phylogenetic relationships of the *Melodidogyne paramali* n. sp. within the genus *Meloidogyne* as inferred from Bayesian analysis of the 18S rDNA sequences using the SYM + I + G model (ln L = −9,656.5775; freqA = 0.2500; freqC = 0.2500; freqG = 0.2500; freqT = 0.2500; R(a) = 1.1490; R(b) = 2.9493; R(c) = 1.9703; R(d) = 0.5777; R(e) = 4.7703; R(f) = 1.0000; Pinva = 0.4140; Shape = 0.4400). Posterior probabilities are given in clades node. Newly obtained sequences are indicated in bold and the sequence codes are given in specimen-clone.

### Meloidogyne marylandi

(Fig. 5)

## Measurements

Measurements of J2 are presented in Table 2.

**Table 2:** Morphometrics of J2 of Meloidogyne marylandi and other populations. All measurements are in micrometer and in the form: mean ± s.d. (range).

	Japan on Podocarpus macrophyllus	Japan on Panicum crus-galli^a^	Israel on Avena strigosa^b^	USA on Zoysia japonica^c^
*n*	14	100	-	20
L	381.7 ± 20.7	392.3 ± 22.05	442.9 ± 13.5	395.1 ± 12.5
	(351.7–418.4)	(338.6–449.3)	(396.0–445.5)	(367.8–411.8)
Body width	13.9 ± 0.6	15.7 ± 0.44	14.8 ± 0.8	16.2 ± 0.8
	(12.8–14.7)	(14.2–16.6)	(13.4–15.9)	(14.8–17.7)
Stylet length	12.5 ± 0.6	12.9 ± 0.45	11.0 ± 0.4	10.8 ± 0.3
	(11.5–13.4)	(11.5–14.1)	(10.4–11.9)	(10.6–11.2)
Stylet base to head	14.5 ± 0.4 (13.7–15.0)	15.4 ± 0.46 (13.7–16.6)	14.6 ± 0.7 (13.4–15.9)	– –
DGO	3.1 ± 0.3	2.5 ± 0.26	2.6 ± 0.4	2.4 ± 0.1
	(2.8–3.6)	(1.8–3.1)	(2.2–2.9)	(1.8–2.9)
Head end to metacorpus valve	55.3 ± 2.4 (51.0–57.9)	45.7 ± 7.3 (30.1–66.3)	52.8 ± 1.9 (51.2–54.9)	51.3 ± 1.4 (49.5–54.3)
Tail length	52.7 ± 1.5	63.7 ± 2.73	63.1 ± 3.1	57.9 ± 1.6
	(49.5–54.0)	(53.6–70.5)	(60.4–69.3)	(55.5–60.2)
Tail hyaline portion	11.2 ± 1.2 (9.5–13.5)	12.3 ± 1.1 (9.3–14.7)	12.4 ± 0.9 (9.9–13.4)	11.5 ± 0.6 (10.0–12.4)
a	27.4 ± 1.2	25.0 ± 1.3	28.8 ± 1.7	24.5 ± 1.0
	(26.4–30.2)	(22.3–28.4)	(26.2–31.3)	(22.9–26.4)
c	7.2 ± 0.3	6.2 ± 0.29	6.8 ± 0.4	6.8 ± 0.2
	(7.0–7.9)	(5.5–7.1)	(6.3–7.6)	(6.4–7.3)

^a^Araki, 1992. ^b^Oka et al., 2003. ^c^Jepson and Golden, 1987.

## Description

### Second-stage juvenile (J2)

Fourteen individuals were measured and observed. Their bodies were vermiform, almost straight, and tapering at both extremities, but tapered more posteriorly. The head was not offset, and was observed with labial disk, while the lip region was without annulation. The stylet was delicate (11.5 μm to 13.4 μm long). The cone was straight, narrow, and sharply pointed; the shaft was observed to become slightly wider posteriorly, with a small rounded knob.

The cuticular annulation was fine and distinct. The lateral field was prominent, with four incisures. The excretory pore was three to four annuli anterior to the hemizonid. The rectum was inflated, while the phasmids were small and indistinct. The tail tapered gradually, and was 49.5 μm to 54.0 μm long. The hyaline tail terminus was 11.2 (9.5–13.5) μm long, and the terminus was bluntly rounded.

Males and females were not found.

## Identification

Means and ranges of all the measurements of J2 (Table 2) of the Japanese population of *M. marylandi* overlapped with those of other Japanese, Israeli, and USA populations of the nematode. The morphological characteristics of J2 (hyaline) tail shape, hemizonid position, and female stylet knob shape were in agreement with the original and additional descriptions (Jepson and Golden, 1987; Araki, 1992; Oka et al., 2003), except that the tail length was slightly shorter.

### Molecular profiles and phylogenetic status

The partial 18S region, full length ITS, 28S D2-D3 region, and the cytochrome oxidase subunit II (CO II) sequences of *M. paramali* n. sp. were deposited in GenBank with the accession numbers MW342769-MW342772, MW342760-MW342768, MW342773-MW342775, and ON430646-ON430649, respectively. The full length ITS and 28S D2-D3 region sequences of *M. marylandi* were deposited in GenBank with the accession numbers ON453657-ON453659 and ON453660-ON453661.

The 18S phylogenetic tree demonstrated that *M. paramali* n. sp. clustered with *M. mali* as an independent clade and close to the basal branch of *Meloidogyne* with high support value (posterior probability = 100). The intraspecific sequence divergence value of the four acquired 18S sequences of *M. paramali* n. sp. is 0.23% to 1.45% (5– 22/1,724 bp). *M. paramali* n. sp. shares the smallest sequence divergence value of 2.58% to 2.64% (41– 45/1707 bp) with *M. mali*.

The ITS phylogenetic tree demonstrated the new species clusters with *M. mali* and *M. vitis* as an independent clade with fully supported value (posterior probability = 100). The new species showed a sister phylogenetic relationship with *M. mali* with low supported value (posterior probability = 60).

The intraspecific sequence divergence value of the nine acquired *M. paramali* n. sp. ITS sequences is 0.15% to 7.35% (1–48/653 bp). The new species also shares the smallest sequence divergence value of 11.86% to 18.80% (81/683–129/686 bp) and 17.85% to 19.70% (108/605–120/609 bp) with *M. mali* and *M. vitis*, respectively.

The 28S D2-D3 phylogenetic tree also displayed a similar topology with the ITS tree. The new species clustered with *M. mali*, an unidentified species *Meloidogyne* sp. GKL-2016, and *M. vitis* as a clade with fully supported value (posterior probability = 100). However, the new species is sistered to the clade that is comprised of *M. mali*, *M. vitis*, and *Meloidogyne* sp. GKL-2016. The analysis of the three acquired sequences of *M. paramali* n. sp. showed the intraspecific sequence divergence value of 3.24% to 6.35% (25–49/772 bp). *M. paramali* n. sp. shares the smallest sequence divergence value of 5.96% to 9.46% (46/772–74/782 bp) with *M. mali*. Further, the new species shared the sequence divergence value of 8.42% to 10.49% (65–71/772 bp) and 8.17% to 9.33% (63/771–72/772 bp) with *Meloidogyne* sp. GKL-2016 and *M. vitis*, respectively.

**Figure 7:**
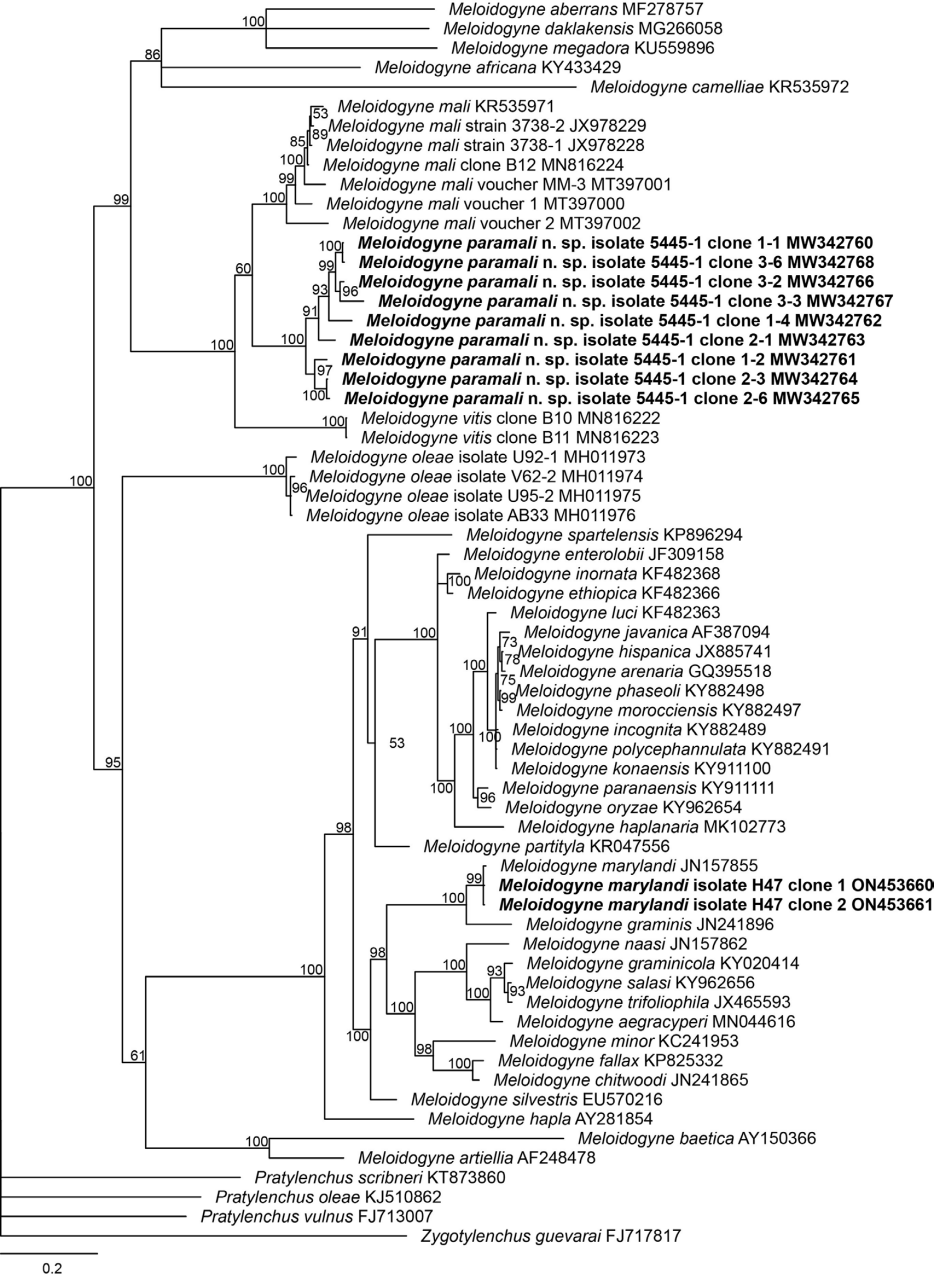
Phylogenetic relationships of the *Melodidogyne paramali* n. sp. within the genus *Meloidogyne* as inferred from Bayesian analysis of the ITS region of rDNA sequences using the GTR+ G model (ln L = –15,611.9209; freqA = 0.2778; freqC = 0.1784; freqG = 0.2084; freqT = 0.3354; R(a) = 1.1160; R(b) = 2.3301; R(c) = 1.4759; R(d) = 0.7128; R(e) = 2.9133; R(f) = 1.0000; Shape = 0.5730). Posterior probabilities are given in clades node. Newly obtained sequences are indicated in bold and the sequence codes are given in specimen-clone.

**Figure 8:**
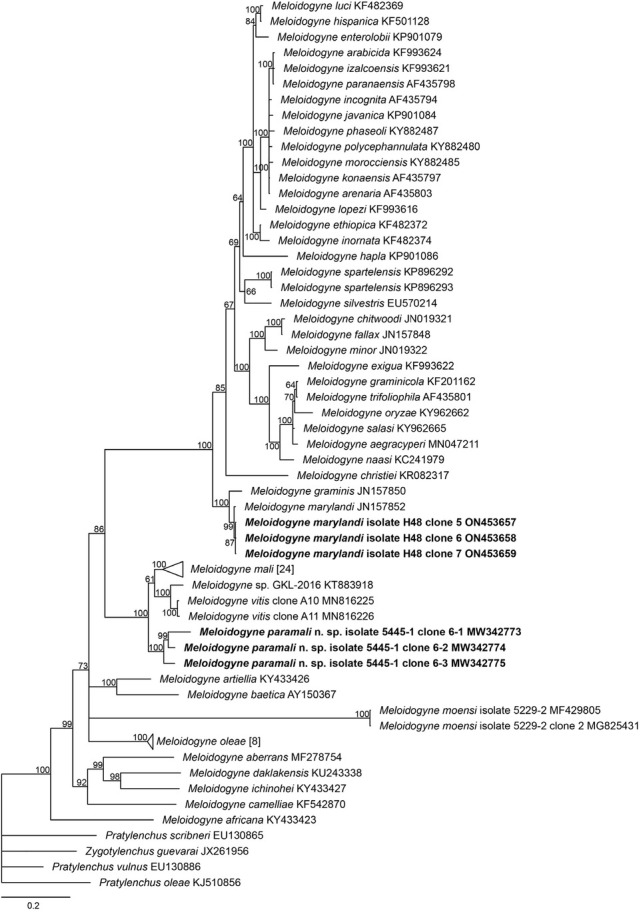
Phylogenetic relationships of the *Melodidogyne paramali* n. sp. within the genus *Meloidogyne* as inferred from Bayesian analysis of the D2–D3 region of the 28S rDNA sequences using the TVM + I + G model (ln L = –9,976.1624; freqA = 0.2157; freqC = 0.1943; freqG = 0.2737; freqT = 0.3163; R(a) = 1.1061; R(b) = 3.8613; R(c) = 1.8155; R(d) = 0.4745; R(e) = 3.8613; R(f) = 1.0000; Pinva = 0.2180; Shape = 0.7340). Posterior probabilities are given in clades node. Newly obtained sequences are indicated in bold and the sequence codes are given in specimen-clone.

**Figure 9:**
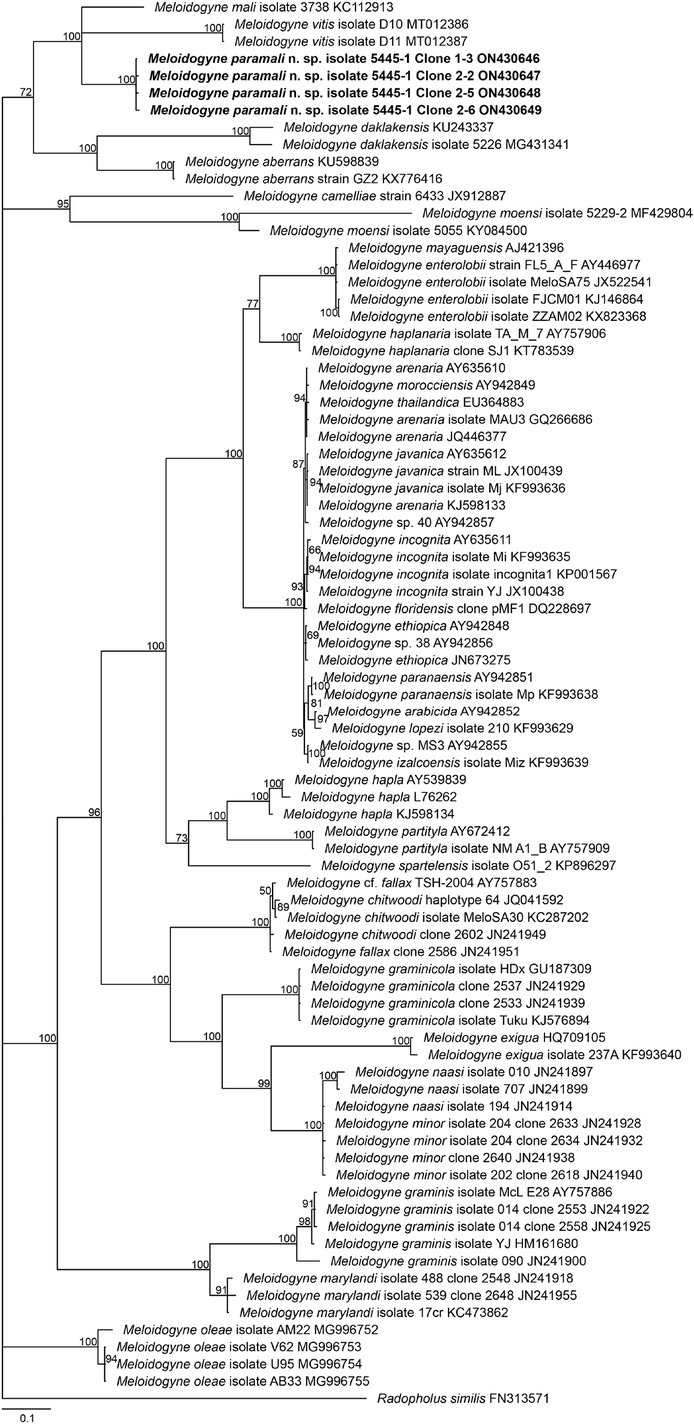
Phylogenetic relationships of the *Melodidogyne paramali* n. sp. within the genus *Meloidogyne* as inferred from Bayesian analysis of the intergenic region between CO II and 16S rDNA sequences using the GTR + G model (ln L = −9,869.8599; freqA = 0.3676; freqC = 0.0265; freqG = 0.0939; freqT = 0.5121; R(a) = 3.2156; R(b) = 5.9094; R(c) = 1.5251; R(d) = 2.6383; R(e) = 13.9890; R(f) = 1.0000; Shape = 0.6340). Posterior probabilities are given in clades node. Newly obtained sequences are indicated in bold and the sequence codes are given in specimen-clone.

In the COII phylogenetic tree, *M. paramali* n. sp. clustered with *M. mali* and *M. vitis* into a fully supported clade (posterior probability = 100). However, the phylogenetic relationship between these three species has remained unresolved. The analysis of the four acquired *M. paramali* n. sp. intergenic regions between CO II and 16S rDNA sequences showed the intraspecific sequence divergence value of 0.14% to 0.44% (1/687–3/688 bp). *M. paramali* n. sp. shares the sequence divergence values of 13.19% to 13.37% (72–73/546 bp) and 18.92% to 19.22% (102/539– 104/541 bp) with *M. mali* and *M. vitis*, respectively. This molecular phylogenetic analysis supports the status of *M. paramali* n. sp. as a new species.

The sequence analysis of ribosomal rDNA 28S and ITS regions showed a 98% to 100% sequence similarity between the *M. marylandi* population from Japan and the registered *M. marylandi*, but it was quite different from other similar species. Therefore, the nematode was identified as *M. marylandi*.

## Discussion

In Japan, the four common nematode species having a major economic ramification are *M. incognita* (Kofoid and White, 1919) Chitwood, 1949, *M. javanica* (Treub, 1885) Chitwood, 1949, *M. arenaria* (Neal, 1889) Chitwood, 1949, and *M. hapla* Chitwood, 1949; and these have already been reported in various hosts (Inagaki, 1985). Besides, *Meloidogyne mali*, *M. suginamiensis*, *M. camelliae* Golden, 1979, and *M. marylandi* have also been reported from Japan, and the former three species may originate from Japan.

In China, non-Chinese *Meloidogyne* species are on the Chinese import plant quarantine list. Therefore, many Japanese landscape trees exported from Japan to China have been quarantine inspected in China. Except the four common species that were often detected from various hosts, *M. mali* was detected from maple (*Acer palmatum* Thunb) and Crape myrtle (*Lagerstroemia indica* L.) (Gu et al., 2013, 2015), *M. camelliae* was detected from *Podocarpus* (*Podocarpus macrophyllus*) and Camellia (*Camellia sasanqua*) (Wang et al. 2013), *M. suginamiensis* was detected from maple tree (Gu et al., 2019), and *M. marylandi* was detected in this report from *Podocarpus* (the host was not confirmed, because the female was not detected from root of *Podocarpus*; and thus, the host may be a kind of grass grown near the tree). Among them, *M. mali* and *M. camelliae* may be widely distributed species in Japan because they have been detected hundreds of times, while *M. marylandi*, *M. suginamiensis*, and *M. paramali* n. sp. are rare, since they were detected only once among thousands of samples.

*M. mali* was first reported in Japan in 1969, with the type host being apple (*Malus pumila* Mill) (Itoh et al. 1969), and has caused significant damage by inducing root galls on its host plant and by reducing host growth by interfering with water and nutrient uptake. *M. mali* has a wide host range, including trees, shrubs, and herbaceous plants. *M. mali* is considered to have been introduced first into the Netherlands as part of a breeding program focused on resistance to Dutch elm disease, when large amounts of elm (*Ulmus chenmoui*) material (seeds, cuttings, and occasionally rooted material) were imported from Japan. Now it has an established presence in three European Plant Protection Organization (EPPO, 2017) regions: France, Italy, and the Netherlands (http://www.eppo.int/QUARANTINE/Pest_Risk_Analysis/PRA_intro.htm). In 2016, *M. mali* was first reported from a declining hedge of Manhattan Euonymus (*Euonymus kiautschovicus* Loes.) growing at a private residence in Harrison, NY, USA. And in 2019, it was first reported from elm trees in the UK. So, its distribution in Asia, Europe, and North America might be wider than originally thought. With the global trade of seedlings and plants, the spread of *M. mali* would pose a great risk. It was added to the EPPO Alert List in 2014 (https://www.eppo.int/QUARANTINE/Alert_List/nematodes/Meloidogyne_mali.htm).

*M. suginamiensis* was first reported on mulberry (*Morus* spp.) from Japan in 1984 (Toida and Yaegashi, 1984), and its hosts include woody plants like maples, mulberries, fig (*Ficus carica* L.), elm (*Ulmus* spp.), raspberry (*Rubus* spp.), and cherry (*Prunus* spp.), and also some weeds and vegetables. *M. suginamiensis* is only reported in Japan, which is very similar to the situation observed in the case of *M. mali*. In 1978, Toida and his colleagues found that a root-knot nematode regarded as *M. mali* from mulberry differed from the species of the original description of *M. mali* in shorter tails of the J2 as well as in the parasitism to herbaceous plants (Toida, 1979). Okamoto et al. (1983) also reported that the population of *M. mali* from Suginami could be distinguished from populations of *M. mali* from apple in Misato, Nagano Prefecture and from mulberry in several localities in the face view of the males and the J2 by SEM observation, tail shape of the J2, and the perineal pattern of the female. However, a conclusive decision on ascertaining a categorization for the newly discovered nematode was not made. Later, Toida and Yaegashi (1984), based on morphological and host plant differences, assigned the name *M. suginamiensis* to the *Meloidogyne* species from mulberry discovered in the small area of Suginami, Tokyo, Japan.

*Meloidogyne camelliae* was first reported from Japanese camellia exported from Japan to USA in 1979, and in 2013 it was also detected from Japanese camellia bonsai imported into Italy. Up to now, it has been reported only in Japan. The exact extent of the damage it inflicts on plants has not yet been fully studied (Golden, 1979; Trisciuzzi et al., 2014).

*M. marylandi* was first reported in the USA, and later on in the republic of Costa Rica, Israel, and Japan, mainly on different species of grasses.

*Meloidogyne* species are spread along long distances mainly through the transportation of plants and seedlings. It is possible that living plants and seedlings with roots could carry dangerous nematodes. International trade has long been recognized as a major pathway by which non-indigenous plant pathogens and pests are moved into new areas (McCullough et al., 2006). The interception of *Meloidogyne paramali* n. sp. and *M. marylandi* in the rhizosphere of maple and *Podocarpus* trees from Japan has emphasized the need for suitable certification programs that produce nematode-free planting material for export to national and international markets. Consequently, accurate diagnosis of the presence of parasitic nematodes is crucial, particularly in the light of detection of *Meloidogyne* species, one of the most dangerous plant parasites recognized to inflict massive damages on crops with disastrous economic consequences, in living plant material intended for export. The potential risk analysis of the new species should be further studied.

The rRNA polymorphism reported in *M. mali* (Gu et al., 2020) is also found in *M. paramali* n. sp. The presence of such character in *M. mali* and *M. paramali* n. sp. suggests the close relationship between these two species, and perhaps that the rRNA polymorphism is widespread across the genus *Meloidogyne*.

Testing of the Koch’s Postulates was not carried out due to an insufficiency in the number of nematodes. *M. paramali* n. sp. is probably a parasite of the maple tree, though the exact extent of damage inflicted is not known. The suitability of *Podocarpus* as a host of *M. marylandi* needs further study.
